# Distribution of phthalate esters and their metabolites in peanut plant during the entire growth period and their dietary risk assessment of peanuts in China

**DOI:** 10.1002/fsn3.4340

**Published:** 2024-07-16

**Authors:** Lixia Fan, Changying Guo, Bingchun Zhang, Mingxiao Ning, Xianfeng Ren

**Affiliations:** ^1^ Institute of Quality Standard and Testing Technology for Agro‐Products Shandong Academy of Agricultural Sciences Jinan China; ^2^ Shandong Provincial Key Laboratory of Test Technology on Food Quality and Safety Jinan China

**Keywords:** accumulation, contamination, exposure risk, metabolism, PAEs

## Abstract

To understand the remediation potential of peanut plants to phthalate esters (PAEs) contamination, the absorption and accumulation patterns of dibutyl phthalate (DBP), bis (2‐ethylhexyl) phthalate (DEHP), and diisononyl ortho‐phthalate (DINP), as well as their metabolites—monoalkyl phthalate esters (MPEs), monobutyl phthalate (MBP), monoethylhexyl phthalate (MEHP), and monoisononyl phthalate (MINP), were examined in peanut plant during the entire growth period. It was found that the amounts of DBP and MBP in peanut plants correlated positively, when the DBP content is high, the MBP content is also high, as well as DEHP and MEHP. Additionally, the root contained the highest overall concentrations of DBP, DEHP, DINP, MBP, and MEHP over the course of the growth cycle. To evaluate PAEs contamination and dietary risk of peanuts in China, 18 PAEs and seven MPEs in 490 peanut samples collected from 17 provinces of China were detected by UPLC‐MS/MS, the detection rate of 18 selected PAE in peanut was 100%. The dietary risk assessment suggested that the general population and high consuming population are not at risk of non‐carcinogenic from the PAEs and MPEs found in peanuts of China. There is no need for the general consumption group to take any precautions against the carcinogenic risk of DEHP, and the high consumption group's carcinogenic risk is also within an acceptable range.

## Graphical abstract

DBP, DEHP, and DINP are mainly accumulated in the root of peanut. There is no carcinogenic or non‐carcinogenic risk by intake of peanuts in China. The non‐carcinogenic health risks of PAEs and MPEs in peanuts are most strongly correlated with DBP + MBP concentrations.

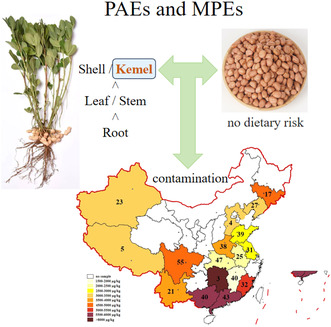

## 1 INTRODUCTION

Plasticizers are a class of synthetic organic compounds widely utilized in industrial applications, with phthalate esters (PAEs) currently being the most extensively employed plasticizers due to their diverse range of uses (Zhang, 2022). Crops can absorb PAEs from surrounding air, water sources, or soil contamination originating from agricultural practices involving plastic film mulch alongside fertilizers or pesticides application (Gao, Liu, et al., 2019; Zhu et al., 2019). Peanut (*Arachis hypogaea*) is an important oil crop. China is the major peanut producer in the world, accounting for about 40% of the global peanut trade (Dong, Wang, et al., 2023). Peanut mulching is mostly used in China. The soil of farmland has been contaminated with different degrees of phthalate due to the long‐term residue of mulch film (Chai et al., 2014; Cui et al., 2013). It has been found that the Σ15PAEs were detected in all the agricultural soils samples of China and the concentration of 15 PAEs was 0.075–6.369 mg·kg^−1^ (Niu et al., 2014). PAEs are a group of highly lipophilic compounds that can migrate from the environment to water and other media, subsequently entering plant tissues (Xia, 2002). PAEs have been detected in various environmental media such as soil, surface water, and air, as well as commonly found in different food sources including cereals and dairy products, indicating their global ubiquity (Arpna & Rajinder, 2021; Cheng, Sun, et al., 2020; Wang et al., 2015).

The toxicity of PAEs and its metabolite monoalkyl phthalate esters (MPEs) has garnered increasing attention within the field of toxicology. PAEs exhibit estrogen‐like effects and reproductive toxicity on humans, leading to their classification as endocrine‐disrupting chemicals (EDCs) by the United States Environmental Protection Agency (US EPA) (Fromme et al., 2011; Gao, Dong, et al., 2019). Additionally, PAEs possess carcinogenic, teratogenic, and mutagenic properties toward human health (Saab et al., 2022; Song et al., 2016). The primary metabolites of DBP, DEHP, and DINP in plants are MBP, MEHP, and MINP (Ema et al., 1995; Ito et al., 2005). Studies have demonstrated that the metabolism of phthalates does not adequately represent the resolution of PAE toxicity; instead, MPEs display stronger biological activity and greater toxicity toward organisms. These effects primarily manifest as male toxicity, female toxicity, and embryonic toxicity (Cheng, Yao, & Sun, 2020; Wang et al., 2013). The accumulation of untransformed PAEs absorbed by plants from the external environment is often the focus, while overlooking the production of MPEs which through metabolic reactions within plants. This oversight underestimates the potential risk of PAEs to human health since MPEs can also pose a threat (Zhang, 2016). Given the widespread presence and potential risks associated with both PAEs and MPEs contamination levels across various food sources, there is a need for the PAE to be investigated for a comprehensive risk assessment approach.

Phytoremediation is one of the means to remove organic pollution. Plants can repair plasticizer pollution through their own absorption and metabolism. Plants can absorb organic compounds from soil interstitial water through their roots; this ability is directly related to octanol–water partition coefficient (Kow) values associated with absorbed organic matter (Burken, 1998). Substances with strong lipophilic properties are more easily absorbed by plant roots (Boxall et al., 2006; Briggs & Evans, 1982). All PAEs are lipophilic substances, and Kow is often used to evaluate the absorption capacity of plants to pollutants, logKow (DBP) = 4.45, logKow (DEHP) = 7.50, logKow (DINP) = 9.40 (Xia, 2002). Most hydrophobic substances undergo chemical transformations after being absorbed by plants (Huang et al., 2009; Mackintosh et al., 2006; Yu et al., 2013). Metabolites derived from these reactions further undergo additional chemical changes leading toward the production of conjugated metabolites (Calderón‐Preciado et al., 2012). The main metabolic pathway of PAEs with carboxylate structure in plants is hydrolysis reaction. The primary metabolite of PAEs is MPEs. When the metabolic reaction of PAEs degradation to MPEs occurs, MPEs can also undergo similar metabolic degradation reactions in plants, leading to the production of other metabolites. Furthermore, the metabolic rate of MPEs is faster than that of PAEs (Sun et al., 2015). Zhu et al., 2019 showed that DBP absorption by rice roots coincided with its metabolism in plants, and the metabolites were mainly MBP and supplemented by phthalic acid (PA).

The purpose of this study was to investigate the accumulation and metabolism of PAEs during the entire life cycle of peanuts in China, and to analyze whether it has the potential of phytoremediation of PAEs. Additionally, it aimed to assess the exposure risk posed by PAEs in peanuts and their products, determine dietary exposure levels among Chinese residents, and provide a basis for food safety supervision and risk management of China.

## 2 MATERIALS AND METHODS

### 2.1 Chemicals and standards

Acetonitrile, methanol, and formic acid (HPLC grade) were purchased from Thermo Fisher Scientific (Waltham, MA, USA). Water was purified with a Milli‐Q treatment system (Millipore, Bedford, MA, USA).

The 18 PAEs investigated in the present study were Dimethyl phthalate (DMP), Diethyl phthahte (DEP), Diallyl phthalate (DAP), Diisobutyl phthahte (DIBP), Dibutyl phthahte (DBP), Bis (2‐methoxyethyl) phthahte (DMEP), Bis (4‐methyl‐2‐pentyl) phthalate (BMPP), Bis (2‐ethoxyethyl) phthalate (DEEP), Dipentyl phthalate (DPP), Dihexyl phthahte (DHXP), Benzyl butyl phthahte (BBP), Bis (2‐n‐butoxyethyl) phthalate (DBEP), Dicyclohexyl phthalate (DCHP), Bis (2‐ethylhexyl) phthalate (DEHP), Diphenyl phthalate (DPHP), Di‐n‐octyl phthalate (DNOP), Diisononyl ortho‐phthalate (DINP), Dinonyl phthalate (DNP). Standards of Monomethyl phthalate (MMP), Monoethyl Phthalate (MEP), Monobutyl phthalate (MBP), Monobenzyl phthalate (MBzP), Monocyclohexyl phthalate (MCHP), Monoethylhexyl phthalate (MEHP), Monoisononyl phthalate (MINP) were purchased from AccuStandard, Inc. (New Haven, USA).

### 2.2 Plant material and treatment

The Huayu No. 22 peanut variety was examined utilizing 300 mm × 300 mm (inner diameter × height) ceramic pots as the culture device. Four treatment groups made up the experiment, the experiment as follows; one was the control (CK), which received no PAE additions, while the other three groups received DBP, DEHP, and DINP additions at soil concentrations of 92 mg·kg^−1^, respectively. Thirteen kilogram of soil which contained N (urea, CH_4_N_2_O) 200 mg·kg^−1^, P [(CaPO_4_)_2_·H_2_O] 150 mg·kg^−1^, K (K_2_SO_4_) 200 mg·kg^−1^ were put into each pot. Each treatment group included five pots in total. Before seeding, the soil was allowed to age for 14 days and the moisture content was adjusted to 70% of the field water capacity. Full seeds of uniform size were selected and evenly distributed in each pot, with three holes made in each pot and two seeds placed in each hole. This ensured that each hole had a strong seedling after emergence. Throughout the experiment, irrigation was done on a regular and quantitative basis. At four growth stages—seedling (M), flowering (H), pod (J), and maturity (C)—three peanut plants from each treatment (CK, DBP, DEHP, DINP) were randomly selected. After the entire plant was dried, the root, stem, leaves, shell, and kernel were separated and stored at −18°C.

### 2.3 Sample preparation

The root, stem, leave, shells, and kernel of peanuts were ground and homogenized. 0.5‐g samples were weighted into a glass centrifuge tube and 4 mL acetonitrile was added, vortex mixed for 1 min, and then ultrasonic extraction for 20 min. After centrifuge at 1664 g for 5 min, the supernatant was taken to blow with nitrogen to near dry at 40°C, set volume with methanol to 1 mL. A reversed phase BEH C18 column (2.1 × 100 mm, 1.7 μm) for liquid chromatography was manufactured by Waters Corporation (Milford, MA, USA). The solution was filtered and directly injected onto ultraperformance liquid chromatography with tandem mass spectroscopy [UPLC‐MS/MS, Waters Acquity UPLC (Milford, MA, USA) tandem AB5500 triple‐quadrupole tandem mass spectrometer (AB Sciex; Framingham, MA, USA)] for analysis.

### 2.4 Determination of phthalates

This was carried out according to previous analytical methods (Hu et al., 2012; Tang et al., 2020; Xu et al., 2014) with modifications. The 18 PAEs and 7 MPEs were determined using UPLC‐MS/MS. Mobile phase A consisted of deionized water containing 0.1% formic acid, and mobile phase B consisted of methanol. The gradient steps are shown in Table S1 and the mass spectrometer parameters are shown in Table S2. The declustering potentials and collision energies of the 18 PAEs and seven MPEs (Table S3) were optimized in Analyst software 1.6.2 (AB Sciex).

### 2.5 Dietary risk assessment

A total of 490 peanut samples from 17 provinces of China were collected in 2021. The use of plastic containers and products was avoided during this study to prevent the introduction of phthalate contamination in the samples. All samples were stored in glass bottles at −18°C prior to analysis. The methods of peanut sample treatment and detection are the same as those in Sections 2.3 and 2.4, respectively. In this study, non‐cancer hazard quotient (HQ) and excessive cancer risk (ECR) were examined to assess the risk of daily intake of peanuts in adults. When the population is exposed to two or more pollutants, the total hazard index (HI) is used to evaluate the non‐carcinogenic health risks of various chemical pollutants (Fan et al., 2020), the calculation equation was as follows (Amiridou & Voutsa, 2011):

(1) EDI=C·Q/bw·r

(2) HQ=EDI/RfD

(3) HI=ΣHQ

(4) ECR=SF×EDI

where EDI (estimated daily intake, ng·g^‐1^ body weight/day) is the estimated daily intake of the population from the diet, and *C* is the concentration of PAEs in the peanut sample, if the concentrations are below the limit of quantitation (LOQ), it is calculated as half of the LOQ. *Q* (g·day^‐1^, g·day^‐1^ for short) is the average daily intake, bw (body weight, kg) is the body weight. RfD is reference dose, SF is carcinogenic slope factor. The parameters were set as follows: The consumption of peanuts and their products by general consumers is based on the “One of the Nutrition and Health Monitoring Reports of Chinese Residents: Dietary and Nutrient Intake Status 2010‐2013”, in which the consumption of peanuts and peanut butter is replaced by the consumption of nuts (3.7 g·day^‐1^). The consumption of peanuts and its products among high‐consumption residents was based on the consumption data in the Fifth China Total Diet Study (72.78 g·day^‐1^). Average bw = 63 kg (Dong, Chen, et al., 2023). RfD_DBP_ = 10 μg·(kg·day)^−1^ (EFSA, 2005), RfD_DIBP_ = 100 μg·(kg·day)^−1^ (CSTEE, 1998), RfD_DEP_ = 5000 μg·(kg·day)^−1^ (Sekizawa et al., 2003), RfD_DEHP_ = 50 μg·(kg·day)^−1^ (EFSA, 2005), RfD_DINP_ = 150 μg·(kg·day)^−1^ (Li, 2018). The SF of DEHP exposed orally was 0.014 (kg·day)/mg.

The @RISK software was used to fit the PAEs concentration in peanuts, and the PAEs concentration fitting was taken as the input variable. The non‐carcinogenic health risks and carcinogenic health risks of exposure to nine PAEs in peanuts were used as output variables, and the model was run for 300,000 iterations. When HQ ≥1, there is a potential non‐carcinogenic risk. Currently, DEHP is the only possible human carcinogen in PAEs. According to the US EPA, the carcinogenic risk level at 10^−6^–10^−4^ is an acceptable risk level, less than 1 × 10^−6^ does not require any action (Deng, 2019; Wang et al., 2015).

### 2.6 Method validation and statistical analysis

The average recovery rate (*R*) and relative standard deviation (RSD) were calculated to analyze the precision. The sensitivity of UPLC‐MS/MS assay was determined by the limit of detection (LOD) [3 times of signal‐to‐noise (S/N) ratios] and the limit of quantification (LOQ) (10 times of S/N ratios). The detailed data for method validation are shown in Table S4. SPSS 22.0 (International Business Machines Corporation, Armonk, New York, USA) was used for data statistical analysis, and @risk 17 software was used for risk analysis.

## 3 RESULTS

### 3.1 Distribution of PAEs and MPEs in peanut tissues

**FIGURE 1:**
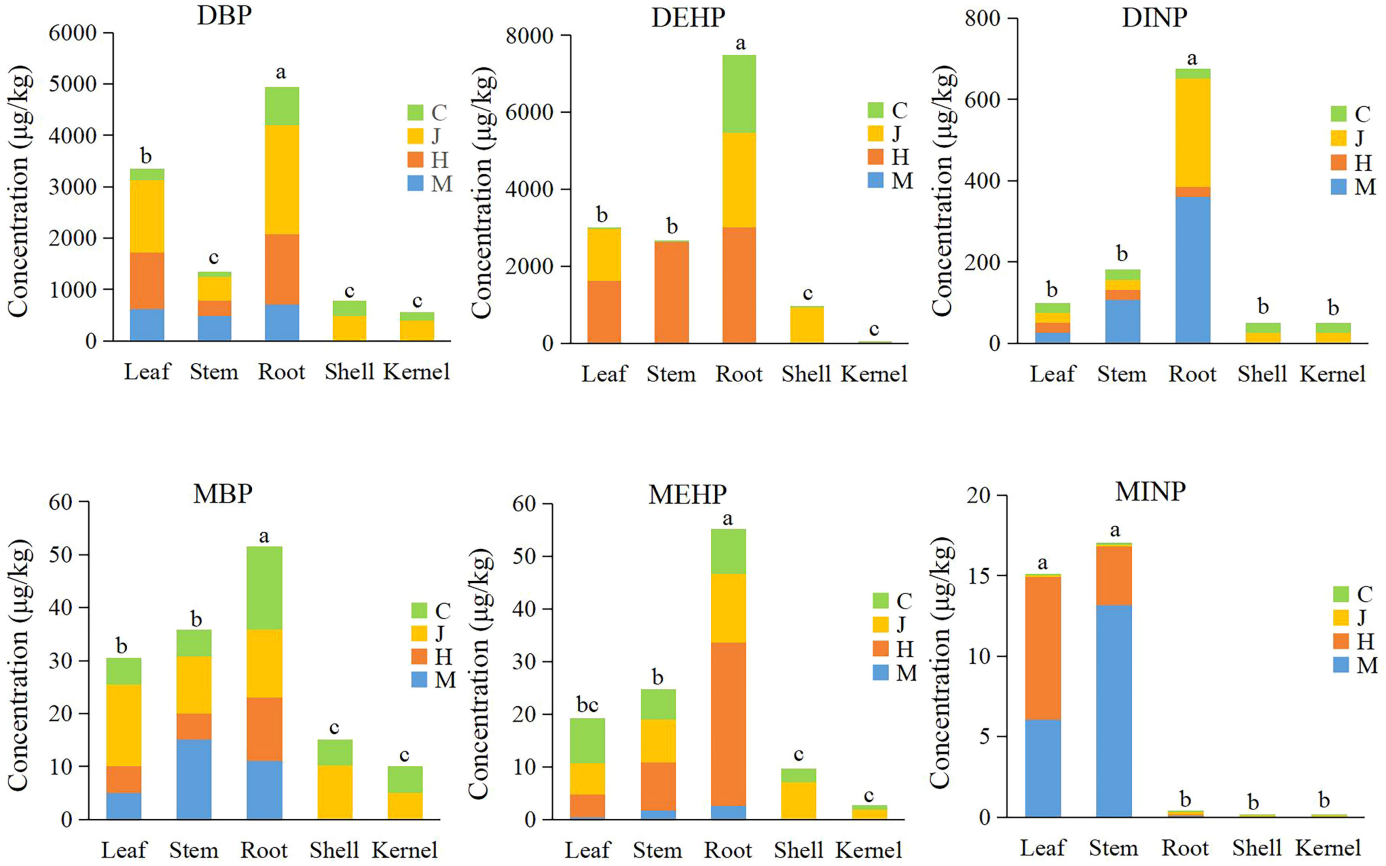
Content of PAEs and MPEs in tissues of peanut at different periods. Different letters on bars denote significant difference at *p* < 0.05 between tissues of peanut.

The amounts and distribution of DBP, DEHP, and DINP as well as their metabolites MBP, MEHP, and MINP in the various tissues of peanuts growing on contaminated soil at different stages are shown in Figure 1. Different tissues showed varying PAEs distribution at different growth stages. Throughout the entire growth cycle, the largest total amounts of DBP, DEHP, and DINP absorbed by roots were significantly higher than that of other tissues, with DINP < DBP < DEHP; MBP, MEHP, and MINP were found at low levels. The overall amount of DBP, DEHP, and DINP in peanut tissues was at its maximum at the pod stage, flowering stage, and seedling stage, respectively. MPEs and PAEs follow a similar trend.

### 3.2 Correlation analysis of PAEs and its metabolite MPEs

**FIGURE 2:**
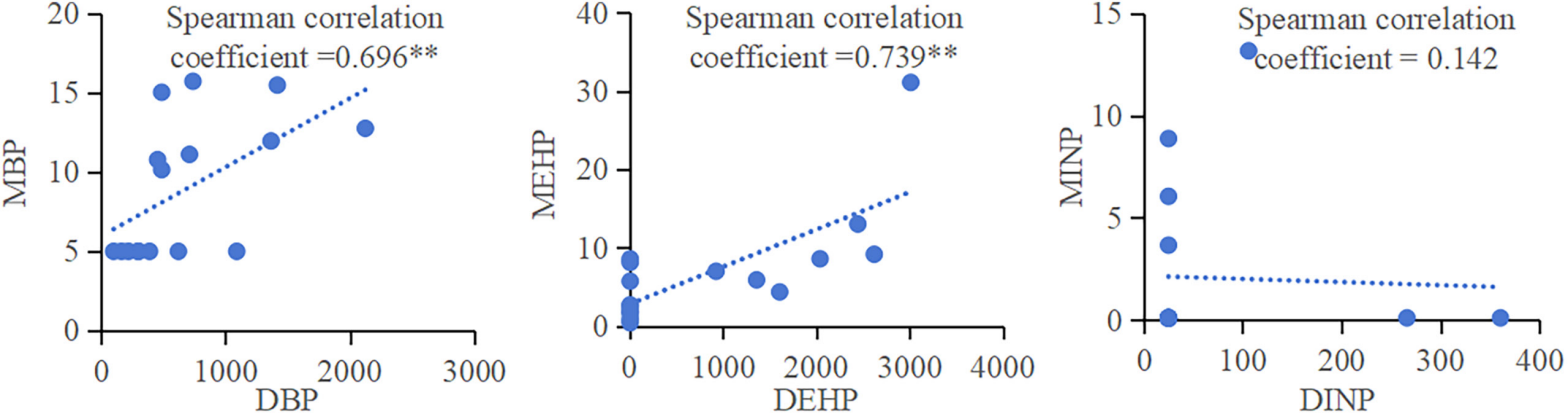
Correlation of DBP‐MBP, DEHP‐MEHP, DINP‐MINP in peanut plant (**Correlation was significant at the 0.01 level).

DBP, DEHP, DINP, and their metabolites MBP, MEHP, and MINP were detected in different tissues of peanut (leaf, stem, root, shell, and kernel) to varying degrees. Therefore, this study carried out correlation analysis of PAE and its metabolite MPE in each group, as shown in Figure 2. The concentrations of DBP–MBP, DEHP–MEHP in peanut plants were significantly positively correlated (*p* < 0.01). DINP and MINP do not show correlation may due to the large number of values below the detection limit.

### 3.3 Overall characteristics of PAEs and MPEs contamination in peanuts

Based on the analysis of 490 samples of peanuts, the statistical data of PAEs and MPEs are shown in Table 1. Of the 18 PAEs and 7 MPEs, the detection frequencies of DEP, DIBP, DBP, DBEP, DEHP, DINP, MBP, MEHP, and MINP were 80.0%, 80.8%, 85.9%, 74.7%, 25.5%, 32.2%, 99.2%, 79.0%, and 31.4%, respectively. The content of PAEs and MPEs in peanut from high to low is: DIBP > DBP > MBP > DINP> DEP > DEHP> MEHP> MINP> DBEP, indicating that the proportion of isomers DIBP and DBP in PAEs contained in peanut was the largest. The concentrations of the other 12 PAEs and 4 MPEs were all below their detection limits and will not be discussed further. The exceedance rate of DBP was the highest, reaching 79.4%, DEHP and DINP did not exceed the standard, and other PAEs did not make limits. The total concentration of 18 PAEs in peanut was not detected ~15133 μg·kg^−1^, the mean was 3600 μg·kg^−1^, and the detection frequency was 100%. The total concentration of the 6 PAEs recommended by EPA for priority control (DMP, DEP, DBP, DNOP, DEHP, and BBP) ranged from not detected (nd) to 7183 μg·kg^−1^, with an average value of 1716 μg·kg^−1^, and the detection frequency was 95.3%.

**TABLE 1:** Statistical results of phthalate plasticizer content in peanut.

	Mean (μg·kg^−1^)	SD	Min (μg·kg^−1^)	Max (μg·kg^−1^)	Detection rate%	LOQ (μg·kg^−1^)	MRL (μg·kg^−1^) (Chanyuan Huang & Mo, 2014)	Over standard rate%
DBP	1657	1522	<LOD	6971	85.9	100	300	79.4
DMP	<LOD	/	<LOD	<LOD	0	20		
DEP	32.5	37.5	<LOD	245	80.0	5		
DEHP	26.7	72.1	<LOD	1141	25.5	10	1500	0
BBP	<LOD	/	<LOD	<LOD	0	0.2		
DNOP	<LOD	/	<LOD	<LOD	0	3		
Σ_6_	1716	1534	‐	7183	95.3			
DAP	<LOD	/	<LOD	<LOD	0	50		
DIBP	1779	1697	<LOD	8133	80.8	100		
DMEP	<LOD	/	<LOD	<LOD	0	0.1		
BMPP	<LOD	/	<LOD	<LOD	0	1		
DEEP	<LOD	/	<LOD	<LOD	0	1		
DPP	<LOD	/	<LOD	<LOD	0	0.2		
DHXP	<LOD	/	<LOD	<LOD	0	0.2		
DBEP	2.41	9.34	<LOD	142	74.7	0.1		
DCHP	<LOD	/	<LOD	<LOD	0	0.1		
DPHP	<LOD	/	<LOD	<LOD	0	0.1		
DINP	102	195	<LOD	2289	32.2	50	9000	0
DNP	<LOD	/	<LOD	<LOD	0	0.5		
Σ_18_	3600	3153	‐	15133	100			
MMP	<LOD	/	<LOD	<LOD	0	5.0		
MEP	<LOD	/	<LOD	<LOD	0	3.0		
MBP	194	203	<LOD	1054	99.2	10.0		
MBzP	<LOD	/	<LOD	<LOD	0	0.2		
MCHP	<LOD	/	<LOD	<LOD	0	0.2		
MEHP	7.61	51.7	<LOD	1110	79.0	0.1		
MINP	6.88	11.1	<LOD	59.9	31.4	0.2		
Σ_25_	3808	3213	‐	15838	100			

Abbreviation: MRL, maximum residue limit.

**FIGURE 3:**
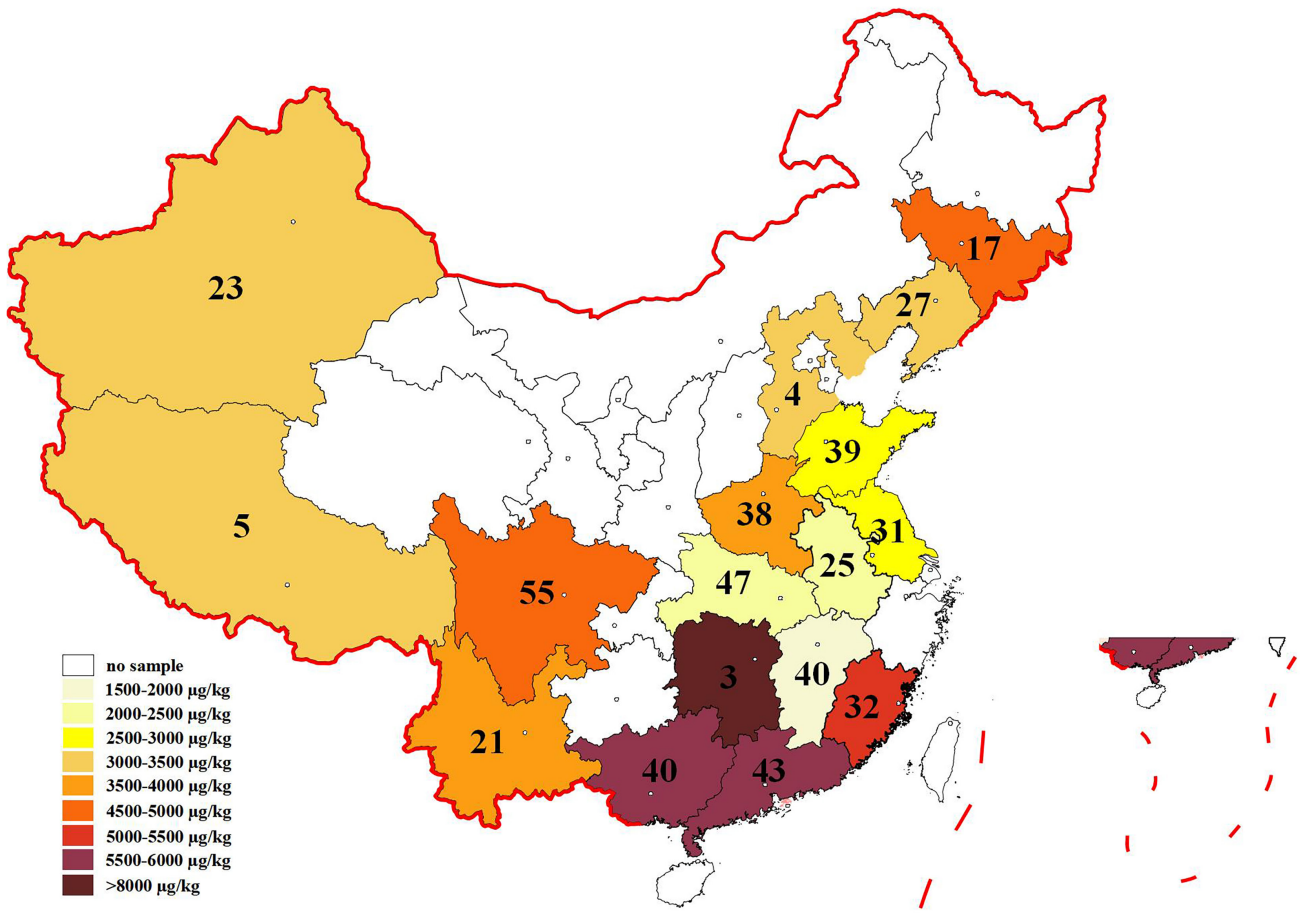
Spatial distribution of Σ25 PAEs and MPEs in peanut of China. The mean concentration of PAEs and MPEs in 65 counties belonging to 17 provinces (Jilin, Liaoning, Hebei, Shandong, Henan, Jiangshu, Anhui, Hubei, Hunan, Sichuan, Jiangxi, Fujian, Guangdong, Guangxi, Yunnan, Xizang, and Xinjiang) of China at 2021. (B) The mean concentration of PAEs and MPEs in five peanut producing regions (Northeast region, North region, Yangtze River drainage area, South region and Northwest region) of China at 2021. The number in the figure shows the number of samples.

To provide a direct insight into PAE and MPE contamination, the spatial distribution of Σ25 PAEs and MPEs in peanuts of China was mapped. As shown in Figure 3, the highest contents of total PAEs and MPEs in peanuts were observed in Fujian, Guangdong, Guangxi, and Hunan provinces, and the average concentration was above 5000 μg·kg^−1^. The total PAE level in northern China is relatively low.

### 3.4 Dietary risk assessment

According to the probability distribution of non‐carcinogenic risk of five PAEs in peanut obtained by Monte Carlo analysis method, the HQ of DBP, DIBP, DEP, DEHP, and DINP in the 97.5 percentile of general peanut consumption population was 3.19 × 10^−2^, 3.60 × 10^−3^, 2.20 × 10^−6^, 1.08 × 10^−4^, and 1.63 × 10^−4^, respectively, all of which were less than 1. The HI of the five PAEs is 3.31 × 10^−2^ < 1 at the 97.5 percentile. The HQ of DBP, DIBP, DEP, and DEHP in the 97.5 percentile of high peanut consumption population was 0.63, 7.95 × 10^−2^, 6.81 × 10^−5^, 4.02 × 10^−3^, and 5.86 × 10^−3^, respectively, which were all less than 1. The HI of the five PAEs is 0.73 < 1 at the 97.5 percentile (Table 2). It can be seen that even in high‐consumption groups, the five PAE in peanuts do not pose a hazard to the human body. Compared with PAEs alone, the HQ of the three PAEs + MAEs all increased to a certain extent, and the HQ of DBP and DBP + MBP of high‐consumption groups had closed to 1, so it is still necessary to attract the attention of high‐consumption people to avoid excessive consumption of peanuts.

**TABLE 2:** Noncarcinogenic risk of PAEs in peanut for adults [μg·(kg·day)^−1^].

HQ	Mean		50th		75th		90th		95th		97.5th	
	General consumption	High consumption	General consumption	High consumption	General consumption	High consumption	General consumption	High consumption	General consumption	High consumption	General consumption	High consumption
DBP	9.75 × 10^−3^	0.19	7.17 × 10^−3^	0.14	1.48 × 10^−2^	0.29	2.31 × 10^−2^	0.45	2.80 × 10^−2^	0.55	3.19 × 10^−2^	0.63
DIBP	1.04 × 10^−3^	2.05 × 10^−2^	7.21 × 10^−4^	1.42 × 10^−2^	1.59 × 10^−3^	3.13 × 10^−2^	2.57 × 10^−3^	5.06 × 10^−2^	3.14 × 10^−3^	6.18 × 10^−2^	3.60 × 10^−3^	7.95 × 10^−2^
DEP	4.24 × 10^−7^	8.35 × 10^−6^	2.01 × 10^−7^	3.96 × 10^−6^	4.59 × 10^−7^	9.02 × 10^−6^	9.61 × 10^−7^	1.89 × 10^−5^	1.49 × 10^−6^	2.94 × 10^−5^	2.20 × 10^−6^	6.81 × 10^−5^
DEHP	2.27 × 10^−5^	4.46 × 10^−4^	9.60 × 10^−6^	1.89 × 10^−4^	1.89 × 10^−5^	3.72 × 10^−4^	3.96 × 10^−5^	7.80 × 10^−4^	6.63 × 10^−5^	1.30 × 10^−3^	1.08 × 10^−4^	4.02 × 10^−3^
DINP	6.49 × 10^−4^	7.04 × 10^−4^	1.70 × 10^−5^	3.34 × 10^−4^	3.21 × 10^−5^	6.31 × 10^−4^	6.40 × 10^−5^	1.26 × 10^−3^	1.03 × 10^−4^	2.03 × 10^−3^	1.63 × 10^−4^	5.86 × 10^−3^
HI	7.47 × 10^−4^	0.21	8.26 × 10^−3^	0.16	1.59 × 10^−2^	0.31	2.42 × 10^−2^	0.48	2.92 × 10^−2^	0.57	3.31 × 10^−2^	0.73
DBP + MBP	1.07 × 10^−2^	0.21	8.35 × 10^−3^	0.16	1.57 × 10^−2^	0.31	2.38 × 10^−2^	0.47	2.88 × 10^−2^	0.57	3.30 × 10^−2^	0.74
DEHP + MEHP	3.30 × 10^−5^	6.49 × 10^−4^	1.28 × 10^−5^	2.53 × 10^−4^	2.56 × 10^−5^	5.04 × 10^−4^	5.45 × 10^−5^	1.07 × 10^−3^	9.26 × 10^−5^	1.82 × 10^−3^	1.55 × 10^−4^	5.91 × 10^−3^
DINP + MINP	3.80 × 10^−5^	7.47 × 10^−4^	1.84 × 10^−5^	3.63 × 10^−4^	3.49 × 10^−5^	6.87 × 10^−4^	6.95 × 10^−5^	1.37 × 10^−3^	1.12 × 10^−4^	2.20 × 10^−3^	1.78 × 10^−4^	6.32 × 10^−3^
HI	2.13 × 10^−2^	0.23	9.51 × 10^−3^	0.19	1.68 × 10^−2^	0.33	2.49 × 10^−2^	0.49	3.00 × 10^−2^	0.59	3.42 × 10^−2^	0.76

**FIGURE 4:**
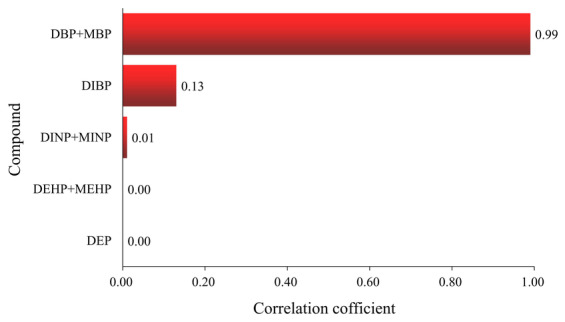
Sensitivity analysis of PAEs non‐carcinogenic risk in peanut.

Spearman correlation analysis was used to generate correlation tornado charts according to correlation order. As shown in Figure 4, the non‐carcinogenic health risk of PAEs and MPEs in peanuts was the most correlated with DBP + MBP concentration, with a correlation coefficient of 0.99, followed by DIBP with a correlation coefficient of 0.13, and other PAEs and MPEs were less correlated with risk.

As DEHP is the only PAEs currently recognized as carcinogenic, this study only assessed the carcinogenic risk of DEHP and DEHP+MEHP (Table 3). The carcinogenic risk of DEHP in the 97.5 percentile of the general consumer population is 7.59 × 10^−8^, and that in the high consumer population is 1.49 × 10^−6^. The carcinogenic risk of DEHP+MEHP in the 97.5 percentile of general consumers is 1.08 × 10^−7^, and these consumers do not need to take any measures; the carcinogenic risk of DEHP + MEHP in the 97.5 percentile of high‐consumption population is 2.13 × 10^−6^, and the carcinogenic risk is also in the acceptable range.

**TABLE 3:** Carcinogenic risk of DEHP and DEHP + MEHP in peanut for adults.

ECR	Mean		50th		75th		90th		95th		97.5th	
	General consumption	High consumption	General consumption	High consumption	General consumption	High consumption	General consumption	High consumption	General consumption	High consumption	General consumption	High consumption
DEHP	1.59 × 10^−8^	3.12 × 10^−7^	6.72 × 10^−9^	1.32 × 10^−7^	1.32 × 10^−8^	2.61 × 10^−7^	2.7719 × 10^−8^	5.46 × 10^−7^	4.64 × 10^−8^	9.13 × 10^−7^	7.59 × 10^−8^	1.49 × 10^−9^
DEHP + MEHP	2.31 × 10^−8^	4.54 × 10^−7^	8.99 × 10^−9^	1.77 × 10^−7^	1.79 × 10^−8^	3.53 × 10^−7^	3.82 × 10^−8^	7.51 × 10^−7^	6.49 × 10^−8^	1.28 × 10^−6^	1.08 × 10^−7^	2.13 × 10^−6^

## 4 DISCUSSION

The remediation mechanisms of PAEs include absorption and metabolism in plants and degradation by microorganisms in their rhizosphere. DINP has a big Kow, long branch chain, and is closely integrated with the root surface of plants, which is difficult to transfer from roots to plants. Therefore, DINP remains at a high level in the soil (Figure S1) and is not easily absorbed by peanut plants. Theoretically, DBP has lower Kow relative to DEHP and DINP, respectively; hence, DBP tend to volatilize or degrade more easily within soil environments. In this study, the lowest content of DBP was found in soil and it was observed that peanuts did not absorb the highest amount of DBP. This could be attributed to either high volatilized degradation of DBP in the environment or neglecting the existence of conjugate metabolites and the degradation reaction of MPEs itself in plants. The content level of MPEs is significantly lower compared to PAEs, which may be due to their conversion into other substances.

The lipid content of plant tissues is positively correlated with the PAEs uptake capacity (Coleman et al., 1997). Among all plant parts, the lipid content of roots is usually significantly higher than that of other parts such as stems and leaves; thus making it easier for lipophilic organic compounds to accumulate there (Dodgen et al., 2013). Consequently, the concentration of accumulated PAEs might be higher in plant roots than other sections. In this study, the total amount of DBP, DEHP, and DINP in peanut roots during the whole growth cycle was significantly higher than that in other tissues such as leaves and stems, indicating that PAEs mainly accumulated in peanut roots. The significant decrease in PAE levels in all parts of peanut during maturity stage suggests that significant metabolism occurs in all tissues. A similar dynamic trend of PAEs first increasing and then decreasing was observed in alfalfa (Ren et al., 2020) and pumpkin seedlings (Lin et al., 2016). The content of metabolite MPEs was not significantly increased, which may be metabolized to other substances, and further studies are needed to verify.

Plant roots can also enhance the biodegradation of PAEs by releasing enzymes and nutrients to promote microbial growth. Peanut, a leguminous plant, can form a symbiotic relationship with nitrogen‐fixing rhizobia, which promotes plant growth and microbial proliferation by increasing the supply of nitrogen in the rhizosphere. Second, the strong root system, large leaf area, and larger aboveground biomass of peanuts allow them to remove more PAEs and may contribute more to restoration. Therefore, peanut may contribute greatly to the phytoremediation of PAEs in soil.

The results of this study showed that the detection rate of six priority control PAEs in peanuts in China reached 95.3%, and the average ∑6 PAE content was 1.72 mg/kg, which was higher than that of Shandong peanuts (Cui, 2014) but lower than that of Nanjing greenhouse vegetables (Wang et al., 2015). In addition, it is important to note that our results showed that the highest detected concentrations of DEHP (1.14 mg/kg) and DBP (6.97 mg/kg) in peanuts exceeded those previously reported in Shandong Province (Cui et al., 2013). Metabolism of PAEs results in intermediates and end products that display different biological activities from their parent compounds (Celiz & Aga, 2009). At present, about dietary risk assessment of the PAEs in agricultural products mainly studied PAE levels; little attention has been paid to the total concentration of PAEs and their metabolite MPEs. In this study, the total hazard index of PAEs + MPEs at the 97.5th percentile in high peanut consumption population increased by 4.1% compared with PAEs alone. This suggests that we should consider the combined risk of maternal and secondary metabolites when evaluating dietary risk, so as to make the evaluation more scientific and reasonable.

## 5 CONCLUSION

This work has shown that different tissues of peanut plant have different degrees of accumulation and metabolism of PAEs. DBP, DEHP, and DINP are mainly accumulated in the root of peanut, and peanut has the potential to remove PAEs from contaminated soil. The non‐carcinogenic health risks of PAEs and MPEs in peanuts are most strongly correlated with DBP + MBP concentrations. The Chinese peanut dietary risk assessment study has determined that there is no carcinogenic or non‐carcinogenic risk by intake of peanuts. There is a lack of mechanistic studies on the response of peanut to PAEs stress at each growth stage, and we hope that these findings can provide valuable information for plant remediation of PAEs‐contaminated soil.

## AUTHOR CONTRIBUTIONS

**Lixia Fan:** Methodology (lead); writing – original draft (lead). **Changying Guo:** Investigation (lead); software (lead). **Bingchun Zhang:** Conceptualization (lead); supervision (lead). **Mingxiao Ning:** Validation (lead); visualization (lead). **Xianfeng Ren:** Project administration (lead); writing – review and editing (lead).

## CONFLICT OF INTEREST STATEMENT

No conflict of interest has been declared by the authors.

## Supporting information


Figure S1

Table S1

Table S2

Table S3

Table S4

## Data Availability

The data that support the findings of this study are available from the corresponding author upon reasonable request.

## References

[fsn34340-bib-0001] Amiridou, D. , & Voutsa, D. (2011). Alkylphenols and phthalates in bottled waters. Journal of Hazardous Materials, 185(1), 281–286. 20933324 10.1016/j.jhazmat.2010.09.031

[fsn34340-bib-0002] Arpna, K. , & Rajinder, K. (2021). Chromatographic methods for the determination of phthalic acid esters in different samples. Journal of Analytical Chemistry, 76(1), 41–56.

[fsn34340-bib-0003] Boxall, A. , Smith, E. , Sinclair, C. , Stutt, E. , & Levy, L. (2006). Uptake of veterinary medicines from soils into plants. Journal of Agricultural and Food Chemistry, 54(6), 2288–2297. 16536609 10.1021/jf053041t

[fsn34340-bib-0004] Briggs, G. , & Evans, A. (1982). Relationships between lipophilicity and root uptake and translocation of non‐ionised chemicals by barley. Pesticide Science, 13(5), 495–504.

[fsn34340-bib-0005] Burken, J. (1998). Predictive relationships for uptake of organic contaminants by hybrid poplar trees. Environmental Science & Technology, 32(21), 3379–3385.

[fsn34340-bib-0006] Calderón‐Preciado, D. , Renault, Q. , Matamoros, V. , Cañameras, N. , & Bayona, J. (2012). Uptake of organic emergent contaminants in spath and lettuce: An in vitro experiment. Journal of Agricultural and Food Chemistry, 60(8), 2000–2007. 22293031 10.1021/jf2046224

[fsn34340-bib-0007] Celiz, M. , & Aga, D. (2009). Pharmaceutical metabolites in the environment analytical challenges and ecological risks. Environmental Toxicology and Chemistry, 28(12), 2473–2484. 19663539 10.1897/09-173.1

[fsn34340-bib-0008] Chai, C. , Cheng, H. , Ge, W. , Ma, D. , & Shi, Y. (2014). Phthalic acid esters in soils from vegetable greenhouses in Shandong peninsula, east China. PLoS One, 9(4), e95701. 24747982 10.1371/journal.pone.0095701 PMC3991724

[fsn34340-bib-0009] Chanyuan Huang, W. C. , & Mo, X. (2014). The toxicity and limited provisions of phthalate esters in food. Packaging and Food Machinery, 32(2), 66–69.

[fsn34340-bib-0010] Cheng, Z. , Sun, H. , Sidhu, H. S. , Sy, N. D. , & Gan, J. (2020). Metabolism of mono‐(2‐ethylhexyl) phthalate in Arabidopsis thaliana: Exploration of metabolic pathways by deuterium labeling. Environmental Pollution, 265, 114886. 32505963 10.1016/j.envpol.2020.114886

[fsn34340-bib-0011] Cheng, Z. , Yao, Y. , & Sun, H. (2020). Comparative uptake, translocation and subcellular distribution of phthalate esters and their primary monoester metabolites in Chinese cabbage (*Brassica rapa* var. chinensis). Science of the Total Environment, 742, 140550. 32623175 10.1016/j.scitotenv.2020.140550

[fsn34340-bib-0012] Coleman, J. , Blake‐Kalff, M. , & Davies, E. (1997). Detoxification of xenobiotics by plants: Chemical modification and vacuolar compartmentation. Trends in Plant Science, 2(4), 144–151.

[fsn34340-bib-0013] CSTEE Ecotoxicity and the Environment (CSTEE) . (1998). Phthalate migration from soft PVC toys and child‐care articles . In: Opinion expressed at the 6th CSTEE plenary meeting, Brussels, 26/27 November, 1998.

[fsn34340-bib-0014] Cui, M. (2014). Pollution risk research of phthalic acid esters in soils and peanuts in Main Peanut producing areas of Shandong Province. Qingdao Agricultural University. (in Chinese).

[fsn34340-bib-0015] Cui, M. , Wang, K. , Wang, L. , & Shi, Y. (2013). Distribution characteristics of phthalic acid esters in soils and peanut kernels in main peanut producing areas of Shandong Province, China. Chinese Journal of Applied Ecology, 24(12), 3523–3530. (in Chinese). 24697074

[fsn34340-bib-0016] Deng, C. (2019). Distribution characteristics and dietary risk assessment of phthalates and their metabolites in porcine tissues. Northeast Agricultural University. (in Chinese).

[fsn34340-bib-0017] Dodgen, L. , Li, J. , Parker, D. , & Gan, J. (2013). Uptake and accumulation of four PPCP/EDCs in two leafy vegetables. Environmental Pollution, 182, 150–156. 23911624 10.1016/j.envpol.2013.06.038 PMC3910503

[fsn34340-bib-0018] Dong, W. , Chen, C. , Zhao, X. , Yang, Y. , Zhao, J. , Xiao, P. , & Chu, Z. (2023). Contamination status and dietary exposure assessment of aflatoxin B1 in peanut and its products sold in Shandong province. China Oils and Fats, 48(7), 67–72. (in Chinese).

[fsn34340-bib-0019] Dong, Y. , Wang, L. , Cai, D. , Zhang, C. , & Zhao, S. (2023). Risk assessment on dietary exposure to aflatoxin B1, heavy metals and phthalates in peanuts, a case study of Shandong province, China. Journal of Food Composition and Analysis, 120, 105359.

[fsn34340-bib-0020] EFSA . (2005). Opinion of the Scientific panel on food additives, flavourings, processing AIDS and material in contact with food (AFC) on a request from the Commission related to Di‐butylphtalate (DBP) for use in food contact materials .

[fsn34340-bib-0021] Ema, M. , Kurosaka, R. , Amano, H. , & Ogawa, Y. (1995). Developmental toxicity evaluation of mono‐n‐butyl phthalate in rats. Toxicology Letters, 78(2), 101–106. 7618175 10.1016/0378-4274(94)03241-x

[fsn34340-bib-0022] Fan, L. , Chen, L. , Cui, W. , Dong, Y. , Yuan, X. , Wang, L. , Liang, J. , & Zhao, S. (2020). Analysis of heavy metal content in edible honeysuckle (*Lonicera japonica* Thunb.) from China and health risk assessment. Journal of Environmental Science and Health, Part B, 55(10), 921–928. 10.1080/03601234.2020.1797426 32720560

[fsn34340-bib-0023] Fromme, H. , Gruber, L. , Seckin, E. , Raab, U. , Zimmermann, S. , Kiranoglu, M. , Schlummer, M. , Schwegler, U. , Smolic, S. , & Völkel, W. (2011). Phthalates and their metabolites in breast milk — Results from the Bavarian monitoring of breast milk (BAMBI). Environment International, 37(4), 715–722. 21406311 10.1016/j.envint.2011.02.008

[fsn34340-bib-0024] Gao, M. , Dong, Y. , Zhang, Z. , & Song, Z. (2019). Metabolism and distribution of dibutyl phthalate in wheat grown on different soil types. Chemosphere, 236, 124293. 31310966 10.1016/j.chemosphere.2019.07.024

[fsn34340-bib-0025] Gao, M. , Liu, Y. , Dong, Y. , & Song, Z. (2019). Physiological responses of wheat planted in fluvo‐aquic soils to di (2‐ethylhexyl) and di‐n‐butyl phthalates. Environmental Pollution, 244, 774–782. 30388681 10.1016/j.envpol.2018.10.095

[fsn34340-bib-0026] Hu, X. , Xu, X. , Ding, C. , Jin, Y. , & Lin, S. (2012). Simultaneous determination of 11 phthalate metabolites in urine by liquid chromatography tandem mass spectrometry. Journal of Hygiene Research, 41(1), 109–112. 22443069

[fsn34340-bib-0027] Huang, H. , Christie, P. , Wang, S. , & Xie, M. (2009). Behavior of decabromodiphenyl ether (BDE‐209) in the soil‐plant system: Uptake, translocation, and metabolism in plants and dissipation in soil. Environmental Science & Technology, 44(2), 663–667. 10.1021/es901860r 20000822

[fsn34340-bib-0028] Ito, R. , Seshimo, F. , Miura, N. , Kawaguchi, M. , Saito, K. , & Nakazawa, H. (2005). High‐throughput determination of mono‐ and di(2‐ethylhexyl) phthalate migration from PVC tubing to drugs using liquid chromatography‐tandem mass spectrometry. Journal of Pharmaceutical and Biomedical Analysis, 39(5), 1036–1041. 16061340 10.1016/j.jpba.2005.06.016

[fsn34340-bib-0029] Li, H. (2018). Occurrence and risk assessment of 22 phthalate esters in bottled drinking water and dried fruits in leisure food. Hainan University. (in Chinese).

[fsn34340-bib-0030] Lin, Q. , Yang, X. , Huang, X. , Wang, S. , Chao, Y. , & Qiu, R. (2016). Subcellular distribution and uptake mechanism of di‐n‐butyl phthalate in roots of pumpkin (*Cucurbita moschata*) seedlings. Environmental Science and Pollution Research, 23, 329–337. 26304812 10.1007/s11356-015-5247-3

[fsn34340-bib-0031] Mackintosh, C. E. M. J. , Ikonomou, M. G. , & Gobas, F. A. (2006). Sorption of phthalate esters and PCBs in a marine ecosystem. Environmental Science & Technology, 40(11), 3481–3488. 16786683 10.1021/es0519637

[fsn34340-bib-0032] Niu, L. , Xu, Y. , Xu, C. , Yun, L. , & Liu, W. (2014). Status of phthalate esters contamination in agricultural soils across China and associated health risks. Environmental Pollution, 195, 16–23. 25194267 10.1016/j.envpol.2014.08.014

[fsn34340-bib-0033] Ren, W. , Wang, Y. , Huang, Y. , Liu, F. , & Teng, Y. (2020). Uptake, translocation and metabolism of di‐n‐butyl phthalate in alfalfa (*medicago sativa*). Science of the Total Environment, 731, 138974. 32413654 10.1016/j.scitotenv.2020.138974

[fsn34340-bib-0034] Saab, Y. , Oueis, E. , Mehanna, S. , Nakad, Z. , Stephan, R. , & Khnayzer, R. S. (2022). Risk assessment of phthalates and their metabolites in hospitalized patients: A focus on di‐ and mono‐(2‐ethylhexyl) phthalates exposure from intravenous plastic bags. Toxics, 10(7), 357. 35878262 10.3390/toxics10070357 PMC9324282

[fsn34340-bib-0035] Sekizawa, J. , Dobson, S. , & Iii, R. J. T. (2003). Concise international chemical assessment document 52: Diethyl phythalate. WHO.

[fsn34340-bib-0036] Song, X. , Cui, X. , Li, J. , Guo, P. , & Wei, J. (2016). Research advances in soil ecotoxicology of phthalic acid esters (PAEs) exposure. Ecology and Environmental Sciences, 25(11), 1885–1890. (in Chinese).

[fsn34340-bib-0037] Sun, J. , Wu, X. , & Gan, J. (2015). Uptake and metabolism of phthalate esters by edible plants. Environmental Science & Technology, 49(14), 8471–8478. 26090545 10.1021/acs.est.5b01233

[fsn34340-bib-0038] Tang, L. , Jiang, L. , Wang, P. , Lu, L. , Dai, Y. , & Huang, L. (2020). Rapid determination of 21 phthalate esters in beverage by two‐dimensional chromatography‐mass spectrometry. The Food Industry, 41(8), 304–307. (in Chinese).

[fsn34340-bib-0039] Wang, J. , Chen, G. , Christie, P. , Zhang, M. , Luo, Y. , & Teng, Y. (2015). Occurrence and risk assessment of phthalate esters (PAEs) in vegetables and soils of suburban plastic film greenhouses. The Science of the Total Environment, 523, 129–137. 25863503 10.1016/j.scitotenv.2015.02.101

[fsn34340-bib-0040] Wang, J. , Luo, Y. , Teng, Y. , Ma, W. , Christie, P. , & Li, Z. (2013). Soil contamination by phthalate esters in Chinese intensive vegetable production systems with different modes of use of plastic film. Environmental Pollution, 180, 265–273. 23792387 10.1016/j.envpol.2013.05.036

[fsn34340-bib-0041] Xia, F. (2002). Biodegradability research of phthalic acid esters. Zhejiang University.

[fsn34340-bib-0042] Xu, D. , Deng, X. , Fang, E. , Zheng, X. , Zhou, Y. , Lin, L. , Chen, L. , Wu, M. , & Huang, Z. (2014). Determination of 23 phthalic acid esters in food by liquid chromatography tandem mass spectrometry. Journal of Chromatography A, 1324, 49–56. 24326131 10.1016/j.chroma.2013.11.017

[fsn34340-bib-0043] Yu, M. , Liu, J. , Wang, T. , Sun, J. , Liu, R. , & Jiang, G. (2013). Metabolites of 2,4,4′‐tribrominated diphenyl ether (BDE‐28) in pumpkin after in vivo and in vitro exposure. Environmental Science & Technology, 47(23), 13494–13501. 24191731 10.1021/es404144p

[fsn34340-bib-0044] Zhang, T. (2016). Study on the rule of metabolism and accumulation of phthalates and their metabolites in fruits and vegetables. Shanghai Ocean University.

[fsn34340-bib-0045] Zhang, X. (2022). Phthalate esters accumulation in soil and wheat roots and bacterial community changes under mulching film residue. Northwest A&F University.

[fsn34340-bib-0046] Zhu, T. , Du, P. , Zeng, L. , Lü, H. , Zhao, H. , Li, Y. , Mo, C. , & Cai, Q. (2019). Variation in metabolism and degradation of di‐n‐butyl phthalate (DBP) by high‐ and low‐DBP accumulating cultivars of rice (*Oryza sativa* L.) and crude enzyme extracts. The Science of the Total Environment, 668, 1117–1127. 31018452 10.1016/j.scitotenv.2019.03.047

