# Supplementary figures and images for: Distribution of phthalate esters and their metabolites in peanut plant during the entire growth period and their dietary risk assessment of peanuts in China
Source: Food Sci Nutr. 2024 Jul 16;12(10):7202–11. doi: 10.1002/fsn3.4340 (PMC11521647; doi:10.1002/fsn3.4340)

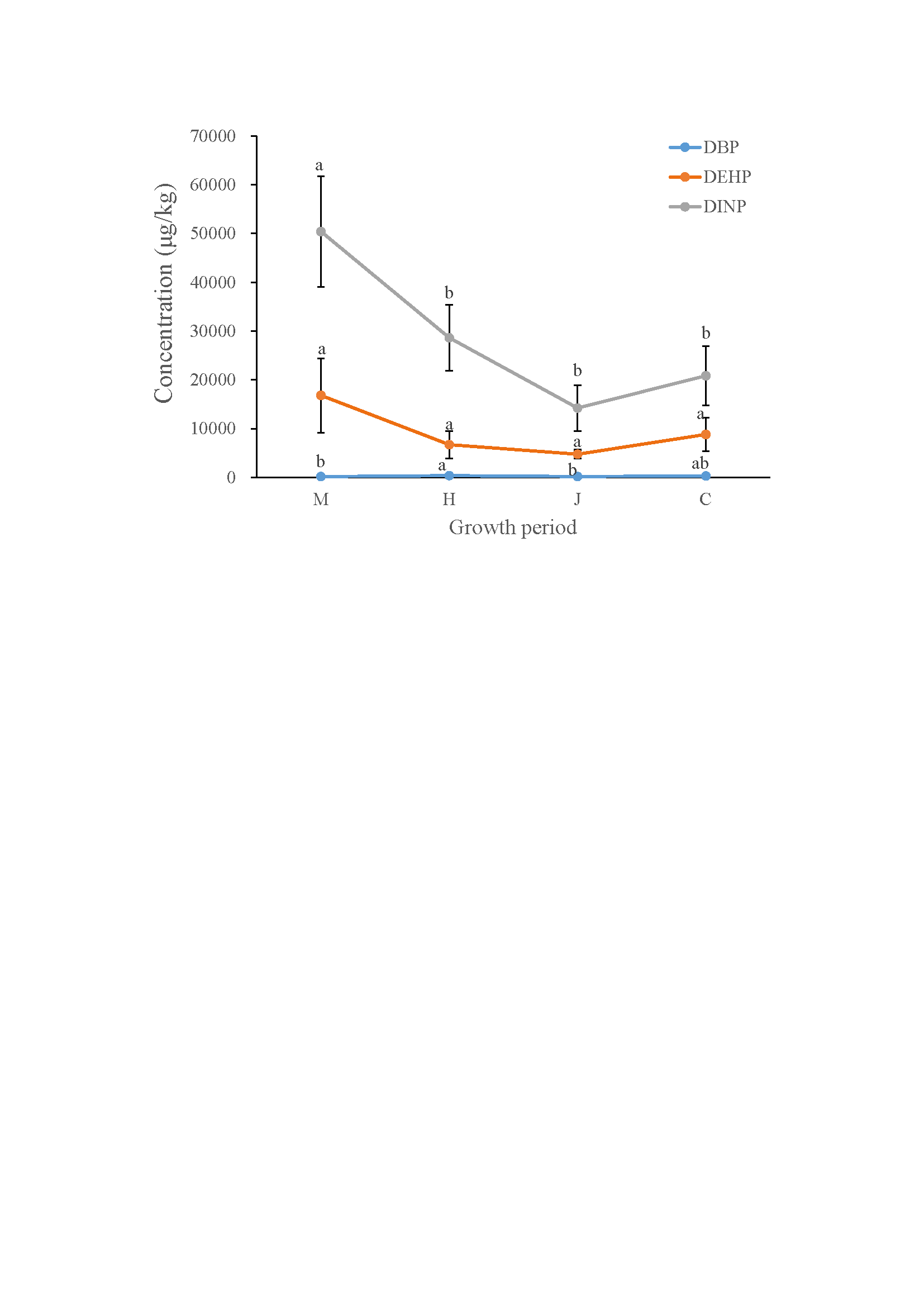

Supplement: Supplementary file 1 — Figure S1 [file FSN3-12-7202-s001.png]
