# Supplementary material for: Distribution of phthalate esters and their metabolites in peanut plant during the entire growth period and their dietary risk assessment of peanuts in China
Source: Food Sci Nutr. 2024 Jul 16;12(10):7202–11. doi: 10.1002/fsn3.4340 (PMC11521647; doi:10.1002/fsn3.4340)
Supplement: Supplementary file 2 — Table S1 [file FSN3-12-7202-s005.docx]

**Table S1**

The gradient steps.

| **Time/min** | **Gradient steps (mobile phase B)** | **Pattern** |
| --- | --- | --- |
| 0～1 | 20%～40% | linearity |
| 1～3 | 40%～60% | linearity |
| 3～5 | 60%～80% | linearity |
| 5～7 | 80%～90% | linearity |
| 7～9 | 90%～95% | linearity |
| 10～13 | 95% | balance |
| 13～13.5 | 95%～50% | linearity |
| 13.5～14 | 50%～20% | linearity |
