# Supplementary material for: Distribution of phthalate esters and their metabolites in peanut plant during the entire growth period and their dietary risk assessment of peanuts in China
Source: Food Sci Nutr. 2024 Jul 16;12(10):7202–11. doi: 10.1002/fsn3.4340 (PMC11521647; doi:10.1002/fsn3.4340)
Supplement: Supplementary file 3 — Table S2 [file FSN3-12-7202-s004.docx]

**Table S2**

The mass spectrometer parameters.

| **Items** | **Parameters** | **Items** | **Parameters** |
| --- | --- | --- | --- |
| column temperature | 35°C | nebuliser gases pressure (gas 1) | 55 psi |
| ion spray voltage | 4.5 kV | Turbo gases pressure (gas 2) | 55 psi |
| curtain gas pressure | 35 psi | turbo heater temperature | 550～600°C |
| mode | MRM |  |  |
