# Supplementary material for: Distribution of phthalate esters and their metabolites in peanut plant during the entire growth period and their dietary risk assessment of peanuts in China
Source: Food Sci Nutr. 2024 Jul 16;12(10):7202–11. doi: 10.1002/fsn3.4340 (PMC11521647; doi:10.1002/fsn3.4340)
Supplement: Supplementary file 4 — Table S3 [file FSN3-12-7202-s002.docx]

**Table S3**

The basic information and MRM parameter of 18 PAEs and 7 MPEs

|  | **Abbreviation** | **CAS number** | **Quantifying ions** | **Qualitative ions** | **Declustering Potential/V** | **Collision energy/V** | **Retention/min** |
| --- | --- | --- | --- | --- | --- | --- | --- |
| Dimethyl phthalate | DMP | 131-11-3 | 195.1/163.1 | 195.1/163.1 | 24 | 16 | 3.76 |
|  |  |  |  | 195.1/133.1 | 24 | 33 |  |
| Diethyl phthahte | DEP | 84-66-2 | 223.2/149.2 | 223.2/149.2 | 16 | 14 | 4.87 |
|  |  |  |  | 223.2/177.1 | 16 | 8 |  |
| Diallyl phthalate | DAP | 131-17-9 | 247.2/65.3 | 247.2/65.3 | 29 | 52 | 5.33 |
|  |  |  |  | 247.2/93.0 | 29 | 42 |  |
| Diisobutyl phthahte | DIBP | 84-69-5 | 279.3/149.0 | 279.3/149.0 | 20 | 19 | 6.59 |
|  |  |  |  | 279.3/93.0 | 20 | 58 |  |
| Dibutyl phthahte | DBP | 84-74-2 | 279.2/149.1 | 279.2/149.1 | 16 | 20 | 6.68 |
|  |  |  |  | 279.2/93.1 | 16 | 57 |  |
| Bis (2-methoxyethyl) phthahte | DMEP | 117-82-8 | 283.2/59.1 | 283.2/59.1 | 66 | 20 | 3.86 |
|  |  |  |  | 283.2/206.9 | 66 | 12 |  |
| Bis (4-methyl-2-pentyl) phthalate | BMPP | 146-50-9 | 283.2/59.1 | 335.3/149.0 | 20 | 10 | 7.96 |
|  |  |  |  | 335.3/167.1 | 20 | 14 |  |
| Bis (2-ethoxyethyl) phthalate | DEEP | 605-54-9 | 311.2/72.9 | 311.2/72.9 | 24 | 16 | 4.88 |
|  |  |  |  | 311.2/221.0 | 24 | 12 |  |
| Dipentyl phthalate | DPP | 131-18-0 | 307.1/149.2 | 307.1/149.2 | 40 | 18 | 7.51 |
|  |  |  |  | 307.1/71.1 | 40 | 23 |  |
| Dihexyl phthahte | DHXP | 84-75-3 | 335.2/149.1 | 335.2/149.1 | 26 | 18 | 8.32 |
|  |  |  |  | 335.2/233.2 | 26 | 12 |  |
| Benzyl butyl phthahte | BBP | 85-68-7 | 313.2/91.2 | 313.2/91.2 | 18 | 14 | 6.58 |
|  |  |  |  | 313.2/149.0 | 18 | 21 |  |
| Bis (2-n-butoxyethyl) phthalate | DBEP | 117-83-9 | 367.2 | 367.2/101.0 | 16 | 16 | 6.66 |
|  |  |  |  | 367.2/249.1 | 16 | 8 |  |
| Dicyclohexyl phthalate | DCHP | 84-61-7 | 367.2/101.0 | 331.3/149.1 | 24 | 16 | 7.58 |
|  |  |  |  | 331.3/167.1 | 24 | 24 |  |
| Bis (2-ethylhexyl) phthalate | DEHP | 117-81-7 | 391.4/149.0 | 391.4/149.0 | 74 | 31 | 9.68 |
|  |  |  |  | 391.4/167.1 | 74 | 15 |  |
| Diphenyl phthalate | DPHP | 84-62-8 | 319.2/225.0 | 319.2/225.0 | 30 | 21 | 6.16 |
|  |  |  |  | 319.2/153.2 | 30 | 35 |  |
| Di-n-octyl phthalate | DNOP | 117-84-0 | 391.4/149.1 | 391.4/149.1 | 61 | 18 | 10.0 |
|  |  |  |  | 391.4/261.2 | 61 | 13 |  |
| Diisononyl ortho-phthalate | DINP | 28553-12-0 | 419.4/149.0 | 419.4/149.0 | 66 | 33 | 10.5 |
|  |  |  |  | 419.4/85.2 | 66 | 23 |  |
| Dinonyl phthalate | DNP | 84-76-4 | 419.4/149.1 | 419.4/149.1 | 68 | 23 | 11.0 |
|  |  |  |  | 419.4/275.3 | 68 | 10 |  |
| Monomethyl phthalate | MMP | 4376-18-5 | 178.5/77.0 | 178.5/77.0 | -12 | -23 | 2.91 |
|  |  |  |  | 178.5/107.0 | -12 | -14 |  |
| Monoethyl Phthalate | MEP | 2306-33-4 | 192.5/76.9 | 192.5/76.9 | -18 | -20 | 3.48 |
|  |  |  |  | 192.5/120.9 | -18 | -15 |  |
| Monobutyl phthalate | MBP | 131-70-4 | 220.9/134.1 | 220.9/134.1 | -19 | -18 | 4.79 |
|  |  |  |  | 220.9/76.9 | -19 | -21 |  |
| Monobenzyl phthalate | MBzP | 2528-16-7 | 254.9/77.0 | 254.9/77.0 | -25 | -17 | 4.81 |
|  |  |  |  | 254.9/183.1 | -25 | -14 |  |
| Monocyclohexyl phthalate | MCHP | 7517-36-4 | 247.0/77.1 | 247.0/77.1 | -18 | -16 | 5.25 |
|  |  |  |  | 247.0/96.9 | -18 | -13 |  |
| Monoethylhexyl phthalate | MEHP | 4376-20-9 | 276.6/134 | 276.6/134 | -30 | -19 | 6.46 |
|  |  |  |  | 276.6/77.1 | -30 | -30 |  |
| Monoisononyl phthalate | MINP | 106610-61-1 | 290.8/77.1 | 290.8/77.1 | -20 | -27 | 6.65 |
|  |  |  |  | 290.8/140.9 | -20 | -24 |  |
