# Supplementary material for: Distribution of phthalate esters and their metabolites in peanut plant during the entire growth period and their dietary risk assessment of peanuts in China
Source: Food Sci Nutr. 2024 Jul 16;12(10):7202–11. doi: 10.1002/fsn3.4340 (PMC11521647; doi:10.1002/fsn3.4340)
Supplement: Supplementary file 5 — Table S4 [file FSN3-12-7202-s003.docx]

**Table S4**

Recovery of PAEs and MPEs (N=3)

| Compound | Recovery (%) | | | LOD (µg/kg) | LOQ (µg/kg) |
| --- | --- | --- | --- | --- | --- |
|  | 100 µg/kg | 200 µg/kg | 400µg/kg |  |  |
| DMP | 91.0±6.8 | 100.3±7.5 | 87.0±6.5 | 20.0 | 50.0 |
| DEP | 88.7±9.8 | 90.5±7.5 | 83.5±5.4 | 5.0 | 10.0 |
| DAP | 71.9±6.9 | 72.1±8.1 | 71.0±3.0 | 50.0 | 100.0 |
| DIBP | 72.3±1.3 | 87.1±12.2 | 70.3±2.9 | 100.0 | 300.0 |
| DBP | 72.0±9.0 | 84.6±7.2 | 81.2±6.8 | 100.0 | 300.0 |
| DMEP | 70.5±4.5 | 76.3±12.7 | 89.1±3.7 | 0.1 | 0.5 |
| BMPP | 72.2±6.0 | 84.0±11.5 | 72.0±5.8 | 1.0 | 2.0 |
| DEEP | 75.9±8.7 | 73.3±13.2 | 90.0±9.8 | 1.0 | 3.0 |
| DPP | 73.3±6.3 | 81.3±9.4 | 85.6±7.7 | 0.2 | 0.5 |
| DHXP | 77.0±5.1 | 71.6±4.1 | 77.0±5.1 | 0.2 | 1.0 |
| BBP | 71.0±6.8 | 70.3±5.0 | 79.0±9.4 | 0.2 | 1.0 |
| DBEP | 71.8±4.8 | 81.5±6.5 | 82.1±7.8 | 0.1 | 0.2 |
| DCHP | 77.6±6.5 | 76.7±8.7 | 90.3±9.3 | 0.1 | 0.2 |
| DEHP | 70.2±1.8 | 71.9±6.1 | 71.7±1.7 | 10.0 | 20.0 |
| DPhP | 70.5±6.4 | 71.9±12.6 | 71.9±2.1 | 0.1 | 0.3 |
| DNOP | 85.0±9.0 | 81.4±8.7 | 82.2±9.4 | 3.0 | 10.0 |
| DINP | 76.6±3.6 | 76.3±4.0 | 72.4±4.6 | 50.0 | 150.0 |
| DNP | 77.0±9.8 | 76.1±2.3 | 78.6±3.2 | 0.5 | 2.0 |
| MMP | 71.7±7.4 | 71.8±3.4 | 73.3±2.7 | 5.0 | 20.0 |
| MEP | 73.7±5.1 | 94.5±9.0 | 96.1±3.0 | 3.0 | 10.0 |
| MBP | 72.3±6.9 | 94.4±5.1 | 99.9±3.2 | 10.0 | 20.0 |
| MBzP | 79.3±7.9 | 90.0±5.3 | 88.2±2.0 | 0.2 | 0.5 |
| MCHP | 79.5±4.0 | 91.4±6.2 | 91.6±3.3 | 0.2 | 0.5 |
| MEHP | 85.0±3.4 | 95.1±5.0 | 90.6±8.6 | 0.1 | 0.2 |
| MINP | 84.4±7.5 | 96.7±6.3 | 93.3±1.2 | 0.2 | 1.0 |
